# Incidence of intraventricular haemorrhage, associated risk factors and short-term outcomes among preterm neonates in a tertiary referral hospital in Kenya

**DOI:** 10.1371/journal.pone.0328406

**Published:** 2025-07-31

**Authors:** Annette Baine Mwatha, Roseline Ochieng, Rosebella Iseme–Ondiek

**Affiliations:** 1 Department of Pediatrics and Child Health, The Aga Khan University Hospital, Nairobi, Kenya; 2 Department of Population Health, The Aga Khan University Hospital, Nairobi, Kenya; Canakkale Onsekiz Mart University School of Medicine, TÜRKIYE

## Abstract

**Background:**

Intraventricular hemorrhage (IVH) stands as the predominant cause of brain injury with the incidence increasing with the decrease in birth weight and gestational age. In developed countries, a reduction in IVH incidence by implementing small baby neuroprotective protocols has been reported. The incidence of IVH within our setting is largely unexplored. This study aimed to fill this gap by determining the incidence and associated risk factors of IVH to guide in implementing a small baby neuroprotective protocol that could potentially reduce the incidence of IVH.

**Method:**

A retrospective cohort study from January 2020 to December 2023 included all Preterm babies with gestation age < 32 weeks or birthweights of ≤1500g admitted to the neonatal intensive care unit (NICU) at The Aga Khan University Hospital, Nairobi. The primary outcome was the occurrence of IVH, while secondary outcomes were associated risk factors and short-term outcomes of IVH.

**Results:**

A total of 526 babies were admitted to the NICU during the study period. Of these, 135 preterm infants were recruited, and 45 (33.3%) developed IVH, predominantly grade 1 IVH, which occurred predominantly between days 0–28 of life. Logistic regression analysis identified that exposure to antenatal steroids exhibited significantly lower odds of IVH occurrence (AOR 0.075, 95% CI 0.007–0.757). Resuscitation in the NICU had a 3 times higher risk of developing IVH (AOR 2.773, 95% CI 0.867–8.874). Treatment with normal saline bolus and inotropes had 4 times higher odds of IVH occurrence (OR 3.5, 95% CI: 1.043–11.885). A higher mortality rate was observed in preterms with IVH (26.6% vs 13.3%). Post-hemorrhagic ventricular dilation (22% vs 6.7%) and periventricular leukomalacia (20% vs 6.7%) were significantly higher among preterms with IVH.

**Conclusion:**

The findings elicited from this study lay a foundation for the implementation of neuroprotective protocols which may potentially reduce the magnitude of IVH in this highly vulnerable age group.

## Introduction

Prematurity ranks second as a leading cause of morbidity and mortality in the neonatal period after intrapartum complications in Kenya, contributing 21 deaths per 1,000 live births to the neonatal mortality rate [1,2]. The burden of prematurity has led to a global movement to achieve Sustainable Development Goal 3.2, aiming to lower the rate of occurrence of premature births and the associated morbidity and death rates of premature newborns [3]. The improvement in maternal services (including Invitro fertilization leading to multiple gestation pregnancies) and newborn intensive care services has caused an increase in the number of preterm deliveries and their survival occurring globally and in sub-Saharan Africa [4]. Consequently, a higher proportion of preterm infants who weigh less than 1500 grams or are less than 32 weeks gestational age (GA) are at risk of experiencing preterm complications, notably acute brain injuries [5–7]. Preterm infants have an immature autoregulatory system and fragile cerebral vasculature which with rapid changes in perfusion of the brain, may cause ischemia or an intraventricular bleed resulting in brain injury. The common forms of acute brain injury in preterm babies include; white matter injury, gray matter injury and intraventricular hemorrhage (IVH) [8,9].

IVH stands as the predominant cause of brain injury occurring in 15–25% of babies born with a birth weight of less than 1500g. This condition poses significant challenges to neurodevelopment and impedes optimal brain growth [10–12]. The incidence of IVH increases with a decrease in birth weight and gestational age [8]. According to the Volpe criteria, IVH is graded according to severity as low/mild (Grade I and II) and high/severe (Grade III and PVHI). IVH grade I is defined as bleeding confirmed to the germinal matrix or germinal matrix hemorrhage plus IVH occupying less than 10% of the lateral ventricular area, grade II is defined as IVH occupying 10–50% of the lateral ventricle area, grade III is defined as IVH occupying greater than 50% of the lateral ventricle area and is associated with acute ventricular dilatation and periventricular hemorrhagic infarction (PVHI) previously referred to as grade IV is defined as hemorrhagic infarction in periventricular white matter ipsilateral to the large IVH [13]. The prevalence of IVH amongst preterm babies <28 weeks gestation age in high-income countries is reported to range from 7–49% and among these 6–22% are grades 3–4 [5]. Studies done in Uganda and Zambia reported a 34.2% prevalence of IVH in preterm babies weighing <2.0 kg with a gestation age of less than 32 weeks. Of these, 19.2% − 54.9% had mild grades and 15% − 27.5% were reported as severe grades [14,15]. In Kenya, a study done in Moi Referral Hospital (2021) reported 67.6% IVH as mild and 32.4% as severe grades amongst preterm infants less than 32 weeks GA [16]. This highlights the magnitude of the occurrence of IVH in both developed and underdeveloped countries.

Most IVH occurs within the first 72 hours after birth (early IVH) and progresses rapidly within the first week of life [17]. This signifies the importance of early identification of the risk factors associated with early IVH to enable the establishment of targeted care bundles in the reduction of IVH [9,18]. The reported risk factors of IVH include low birth weight, low gestational age, perinatal asphyxia, hypothermia, respiratory distress syndrome requiring surfactant treatment, mechanical ventilation, disseminated intravascular coagulation, maternal chorioamnionitis, maternal hypertensive disorders and lack of antenatal steroids [17,19]. Short-term complications of IVH are dependent on the grade of the IVH. IVH grades I and II involve mild bleeding that is often self-limiting while grades III–IV pose a high risk of death or development of post-hemorrhagic ventricular dilation (PHVD) or periventricular leukomalacia [20–22]. Notably, some neonatal critical care units have shown a reduction in the incidence of IVH after implementing small baby protocols to help prevent IVH. Some of the emphasized interventions include antenatal steroids, delayed cord clamping, vitamin K administered IV instead of IM, early empiric caffeine, less aggressive respiratory and hemodynamic support, prophylactic indomethacin and control of sound and light [18].

The frequency of occurrence of IVH within our population is largely unexplored. This information is fundamental for our ability to improve the care of these babies, reducing the morbidity and mortality in this vulnerable population. To implement an IVH care bundle, it is important to have baseline information on the incidence of IVH and its associated risk factors to address each risk factor individually. This study aimed to fill this gap by determining the incidence and associated risk factors of IVH which may be amenable in our neonatal unit setting. In this study, we also determined the grades of IVH, monitored their progress and short-term outcomes in preterm babies less than 32 weeks gestation age or weighing less than 1500g. The baseline information gathered from this study will be used to guide the design and implementation of a small baby neuroprotective protocol that could potentially reduce the incidence of IVH in our neonatal care unit.

## Methodology

### Study design and setting

This retrospective cohort study was conducted in the neonatal intensive care unit (NICU) at The Aga Khan University Hospital Nairobi (AKUHN) from January 2020 to December 2023. As a level 5 hospital (teaching and referral hospital), the NICU at AKUHN receives high-risk babies delivered within the institution, referrals from other hospitals in East and Central Africa or from home. Preterm babies are nursed in humidified incubators and receive respiratory support as per the severity of the respiratory distress. Respiratory support may be rendered via mechanical ventilation, high-flow nasal cannula, continuous positive airway pressure or nasal prongs. Surfactant is administered using INSURE (INtubation- SURfactant- Extubation) to all preterm babies presenting with respiratory distress syndrome (RDS) [23]. All Preterm babies <32 weeks are started on caffeine citrate, empiric antibiotics and receive total parenteral nutrition whilst enteral feeds are introduced and are advanced to full feeds. Cranial ultrasound (US) scans are performed by a qualified radiographer and the findings are validated by a pediatric radiologist. Cranial US scans are routinely done on days 3, 7 and day 14 respectively and thereafter done weekly if there is an intracranial bleed or else the scan is done at day 28 of life. Depending on the condition of the baby, some cranial US scans are done before day 3 of life for example babies noted to have bleeding tendencies and babies presenting with perinatal asphyxia.

### Study population

All preterm neonates with gestation age < 32 weeks or birthweights of ≤1500g admitted to NICU at AKUHN from January 2020 to December 2023 were recruited. Preterm babies with congenital anomalies and those who died before having their first cranial ultrasound scan were excluded.

### Sampling technique and sample size

To achieve the minimum sample size required to meet the objectives, the census sampling technique was used. All preterm infants with <32 weeks gestation age or birthweights of ≤1500g who were admitted to the neonatal unit during the study period and met the eligibility criteria formed the study sample. Assuming that 8% of the preterm neonates had IVH based on the incidence of IVH from the study in Kenyatta National Hospital in Kenya [24], the study would require a minimum sample size of 114 participants to estimate the expected proportion with 5% absolute precision and 95% confidence. Using the Fisher`s formula [25]: N = (Z^2^ x p(1-p))/d ^2^, N = sample size, Z^2^ = the corresponding value to the 95% confidence level (1.96), P = expected proportion of event of interest in the population (8%), d = absolute precision (5%). Further adjusting of sample size by 10% for potential loss due to data collection errors using N = n/(1-d) formula: n = calculated sample size (114), d = drop outrate (0.1), N = 114/0.9 = 127. The estimated sample size for this study was 127 participants.

### Data collection tools

A data retrieval form was used to collect the social demographic data and clinical assessment from both the mothers’ and babies` files.

### Data collection method

Data collection commenced 19^th^ February 2024 and ended 22^nd^ March 2024. Prior to the start of data collection, the data retrieval form was pilot-tested by collecting data from 13 preterm babies which was 10% of the minimum sample size. The data retrieval form was validated by the principal investigator and neonatologist in the unit and a research assistant was trained on the use of the validated data collection tool in data collection. The trained research assistant then retrieved, reviewed and extracted data from the medical records of the babies born during the designated study period. Babies who matched the eligibility criteria had data extracted from their files. The baby`s demographic data and clinical assessment were extracted from the patient file using the data retrieval tool. The mother`s file was also retrieved and reviewed for data extraction. The maternal antenatal records provided information on the demographics of the mother as well as information on intrapartum occurrences such as chorioamnionitis [26], prolonged rupture of membranes (PROM) [27], fever, gestational comorbidities and the use of antenatal steroids. Cranial ultrasounds performed, reported and validated by the Paediatric radiologist on days 0–2, 3, 7, 14–28 were retrieved and categorized as normal or with IVH. According to the Volpe criteria, scans displaying IVH were further categorized as grade I, II, III, or PVHI by the consultant Paediatric radiologist. For every 10 medical records, 5 medical records were randomly selected for quality assessment to compare the data extracted to the original record by the principal investigator.

### Statistical analysis

Data was recorded on paper, entered and cleaned on Excel and then transferred to SPSS (statistical package for social sciences) version 23 for analysis. Categorical variables were presented using frequency and percentages while respective measures of central tendency and measures of spread summarized the numerical variables. Incidence proportion (number of new events divided by the total sample size) was calculated to determine the frequency of occurrence of IVH amongst our study sample. Bivariate analysis using the Chi-square test was undertaken to determine the factors associated with the occurrence of IVH. Logistic regression was used for multivariate analysis to determine the factors independently associated with IVH. A cut–off P-value of 0.25 was used as the entry point for the variables in the univariate analysis to the multivariant analysis. Frequency counts and percentages were used to report the outcomes of IVH amongst preterm babies. Statistical significance was set at the (0.05) 5% level.

### Ethical considerations

Ethical clearance was sought from Aga Khan University research ethics committee Ref: 2024/ISERC-12 (v1) and permission to conduct the research was obtained from the Head of the Department for Neonatology. A research permit Ref No: 402893 was additionally sought from the National Commission for Science, Technology & Innovation (NACOSTI). The Aga Khan University research ethics committee waived the requirement for informed consent since this was a retrospective study of medical records. The principal investigator had access to information that could identify the individual participants upon retrieval of the files from the medical records. Data was coded to declassify it during data collection and patient confidentiality was observed. The principal investigator used a password to electronically encrypt data. Data collected on paper was destroyed after its safe transfer to SPSS version 23. A minimum of 7 years will pass before the electronically stored data is deleted in accordance with The Aga Khan University Hospital data protection guidelines.

## Results

### Background information of study participants

**Fig 1:**
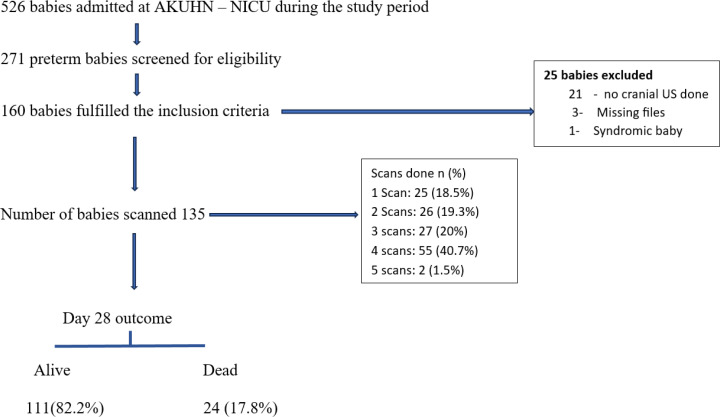
Flow chart of the recruitment process, imaging done, and day 28 outcome. The flow diagram outlines the participant recruitment process, the number of babies scanned (n = 135) and the clinical outcomes assessed at day 28. It also includes the number of excluded study participants and reasons for their exclusion, the number of cranial ultrasounds performed and the outcomes grouped by survival.

A total of 526 babies were admitted to NICU at The Aga Khan University Hospital, Nairobi during the study period from January 2020 to December 2023. Of these, 271 were preterm babies and these were screened for eligibility. One hundred and sixty babies fulfilled the inclusion criteria but 25 babies were excluded because of missing files, no cranial US done and one baby had syndromic features. The final number of scanned preterm babies was 135 Fig 1.

### Social demographic and clinical characteristics of study participants

The demographic and clinical characteristics of the eligible participants are summarized in Table 1. There was a slight predominance of female preterm babies 75(56%) in the study population. Majority of the study participants had birth weights between 1001–1500 grams (46.7%). Majority of the study participants were delivered at a gestational age between 29–32 weeks (73.3%). Majority of mothers delivered via emergency C/S (80%) mainly due to pre-eclampsia and antepartum hemorrhage. The other indications of emergency C/S were oligohydramnios, multiple gestations and abnormal dopplers. Majority of the mothers (63%) received at least 2 doses of antenatal steroids.

**Table 1:** Demographic and clinical characteristics of participants n = 135.

Variable	Category		n (%)		
**Sex**	Male		60 (44.4%)		
	Female		75 (55.6%)		
**Birth weight**	<1000g		46 (34.1%)		
	1000 – 1500g		63 (46.7%)		
	1501 – 2000g		25 (18.5%)		
	2001 – 2500g		1 (0.7%)		
**Gestation age**	<28 weeks		36 (26.7%)		
	29–32 weeks		99 (73.3%)		
**Mode of delivery**	Emergency C/S		108 (80%)		
	Elective C/S		16 (11.9%)		
	SVD		11 (8.1%)		
**Antenatal steroid doses**	No dose		7 (5.2%)		
	1 dose		20 (14.8%)		
	2 doses		84 (62.2%)		
	3 doses		1 (0.7%)		
	6 doses		1 (0.7%)		
	Unknown		22 (16.3%)		
**Resuscitation at birth**	None		25 (18.5%)		
	Oxygen		36 (26.7%)		
	BVM		66 (48.9%)		
	BVM & chest compression		6 (4.4%)		
	Unknown		2 (1.5%)		
**Resuscitation in NICU**	Yes		26 (19.3%)		
	No		108 (80%)		
	Unknown		1 (0.7%)		
**IVH occurrence**	Any IVH		45 (33.3%)		
	No IVH		90 (66.7%)		
**Outcome**	Alive		111 (82.2%)		
	Dead		24 (17.8%)		
**Grade of IVH occurrence**	**Day 0–2 n(%)**	**Day 3** **n(%)**	**Day 7** **n (%)**	**Day 14** **n (%)**	**Day 28** **n (%)**
Grade I	2 (1.5%)	14(10.4%)	21(15.6%)	21(15.6%)	22(16.3%)
Grade II	1 (0.7%)	4 (3.0%)	4 (3.0%)	3 (2.2%)	1 (1.7%)
Grade III	0	1 (0.7%)	1 (0.7%)	1 (0.7%)	0
PVHI	0	1 (0.7%)	2 (1.5%)	4 (3.0%)	4 (3.0%)

**C/S, Cesarean section; SVD, Spontaneous vaginal delivery; BVM, bag-valve-mask; IVH, intraventricular hemorrhage; PVHI, Periventricular hemorrhagic infarction; NICU, Neonatal intensive care unit.**

### Incidence and severity of IVH

**Fig 2:**
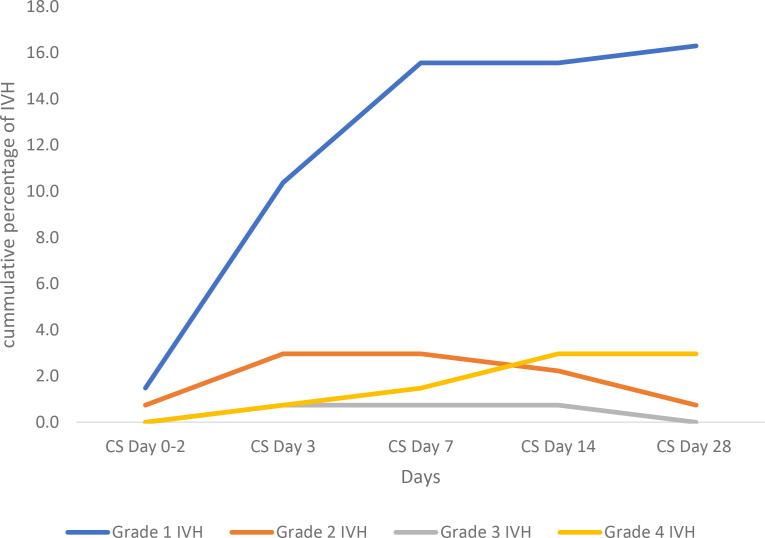
Timing of IVH by grade severity over 28 days of life.

Of the 135 study participants, 45(33.3%) preterm babies developed IVH. Grade 1 IVH occurred most predominantly during the first 28 days of life as shown in Table 1. Fig 2 illustrates the timing and severity of IVH in percentages across different days, categorized by grades. In the early period (Day 3), Grade 1 IVH was prevalent, affecting 10.4% of cases, followed by Grade 2 IVH at 3.0%. As days progressed, the incidence of Grade 1 IVH steadily increased, reaching 16.3% by Day 28. However, Grade 2 IVH steadily decreased over time to 0.7% as observed by Day 28. Grade 3 and PVHI IVH remained relatively low throughout, with Grade 4 IVH slightly increasing from 0.7% on Day 3 to 3.0% by Day 28.

Fig 2. illustrates the cumulative percentages of patients who developed Grade 1 to Grade 4 IVH at different time points. Grade 1 IVH increased most significantly over time, while higher-grade IVH (Grades 2–4) remained relatively rare. The time points shown are Cranial scans (CS) done on Day 0–2, Day 3, Day 7, Day 14, and Day 28. Each line represents the severity of IVH grade, as indicated in the legend.

### Factors associated with the occurrence of IVH

Bivariate analysis showed no difference in the incidence of IVH between male and female preterms. A statistically significant association was observed between the need for NICU resuscitation and the occurrence of IVH (Chi-square test, p = 0.019). Similarly, preterm babies receiving hypotension treatment, particularly inotropes or normal saline (NS) bolus with inotropes, exhibited a significantly higher IVH incidence (Chi-square test, p = 0.012). Preterm babies whose mothers received antenatal steroids exhibited a significantly lower IVH incidence compared to those who were not exposed to antenatal steroids (Chi-square test, p = 0.002). The association between mode of delivery and IVH specifically delivery via emergency cesarean section (Chi-square test, p = 0.069) compared to spontaneous vaginal delivery did not reach the threshold level of significance. Chorioamnionitis, Prolonged rupture of membranes (PROM), Patent ductus arteriosus (PDA), RDS, mechanical ventilation (MV) and platelet counts had no statistically significant associations with IVH occurrence in the bivariate analysis Table 2.

**Table 2:** Bivariant analysis of factors associated with incident IVH.

Variable	Category	Any IVH	No IVH	P-value
**Sex**	**Male**	**22 (36.67%)**	**38 (63.33%)**	**0.462**
	**Female**	**23 (30.67%)**	**52 (69.33%)**	
**Place of birth**	**AKUHN**	**43 (32.33%)**	**90 (67.67%)**	**0.109**
	**Other hospitals**		**2 (100%)**	
**Birth weight**	**<1000g**	**18 (39.13%)**	**28 (60.87%)**	**0.757** ^*^
	**1001 – 1500g**	**19 (30.16%)**	**44 (69.84%)**	
	**1501 – 2000g**	**8 (32%)**	**17 (68%)**	
	**2001 – 2500g**	**0 (0%)**	**1 (100%)**	
**Gestation age**	**<28 weeks**	**16 (44.44%)**	**20 (55.56%)**	**0.099**
	**29–31 weeks**	**29 (29.29%)**	**70 (70.71%)**	
**Mode of delivery**	**SVD**	**7 (63.64%)**	**4 (36.36%)**	**0.069** ^*^
	**Emergency C/S**	**32 (29.63%)**	**76 (70.37%)**	
	**Elective C/S**	**6 (37.50%)**	**10 (62.50%)**	
**Resuscitation at birth**	**Yes**	**37 (34.26%)**	**71 (65.74%)**	**0.356**
	**No**	**6 (24%)**	**19 (76%)**	
**Resuscitation in NICU**	**Yes**	**14 (53.85%)**	**12 (46.15%)**	**0.019**
	**No**	**30 (27.78%)**	**78 (72.22%)**	
**Chorioamnionitis**	**Yes**	**1 (20%)**	**4 (80%)**	**0.556** ^*^
	**No**	**43 (33.33%)**	**86 (66.67%)**	
	**Unknown**	**1 (100%)**	**0 (0%)**	
**PROM**	**Yes**	**15 (40.54%)**	**22 (59.46%)**	**0.304**
	**No**	**29 (29.90%)**	**68 (70.10%)**	
**Hypotension treatment**	**None**	**33 (28.45%)**	**83 (71.55%)**	**0.012** ^*^
	**Inotropes**	**4 (66.67%)**	**2 (33.33%)**	
	**NS bolus & inotropes**	**7 (58.33%)**	**5 (41.67%)**	
	**NS bolus**	**1 (100%)**	**0 (0%)**	
**MV**	**Yes**	**36 (36%)**	**64 (64%)**	**0.267**
	**No**	**9 (25.71%)**	**26 (74.29%)**	
**PDA**	**Yes**	**14 (43.75%)**	**18 (56.25%)**	**0.318**
	**No**	**30 (30.61%)**	**68 (69.39%)**	
**Platelets**	**>150000**	**16 (26.67%)**	**44 (73.33%)**	**0.142**
	**<150000**	**29 (38.67%)**	**46 (61.33%)**	
**ANS**	**Yes**	**29 (27.10%)**	**78 (72.90%)**	**0.002** ^*^
	**No**	**6 (85.71%)**	**1 (14.29%)**	
	**Unknown**	**10 (47.62%)**	**11 (47.62%)**	

* P - Value* - fisher’s exact; ANS, antenatal steroids; PDA, Patent ductus arteriosus; MV, mechanical ventilation; PROM, prolonged rupture of membranes.

Logistic regression analysis was conducted to investigate the factors associated with IVH occurrence. The variables found significant at the univariate level with a cut-off p-value of 0.25 were included in the multivariate analysis. The univariate logistic regression analysis indicated that treatment with NS bolus and inotropes had 4 times higher odds of IVH occurrence (OR 3.5, 95% CI: 1.043–11.885, P = 0.043). EM C/S was significantly associated with a reduced risk of IVH occurrence compared to Spontaneous Vaginal Delivery (SVD) (AOR 0.334, 95% CI 0.066–0.880, p = 0.031). Preterm babies requiring resuscitation in the NICU had a 3 times higher risk of developing IVH however, this association failed to meet the threshold for statistical significance after adjusting for confounders (AOR 2.773, 95% CI 0.867–8.874, p = 0.086). Multivariate logistic analysis indicated that preterm babies exposed to antenatal steroids exhibited significantly lower odds of IVH occurrence (AOR 0.075, 95% CI 0.007–0.757, p = 0.028). z Maternal conditions like chorioamnionitis (AOR 0.5, 95% CI 0.054–4.611, p = 0.541) or PROM (AOR 0.625, 95% CI

0.284-1.374, p = 0.243) showed no significant associations with IVH occurrence Table 3.

**Table 3:** Factors associated with the occurrence of IVH (Primary outcome). Logistic regression analysis.

Variable	Category	COR	P-value	COR (95% CI)	AOR	P-value	AOR (95% CI)
**Sex**	Male	1					
	Female	0.764	0.463	0.37-1.56			
**Mode of delivery**	SVD	1			1		
	Emergency C/S	0.241	0.031	0.066-0.880	0.334	0.167	0.073-1.575
	Elective C/S	0.343	0.187	0.070-1.684	0.562	0.539	0.089-3.537
**Gestation age**	<28	1					
	29–31	0.518	0.101	0.236-1.138	1.821	0.329	0.547-6.064
**Birth weight**	<1000g	1					
	1001 – 1500g	0.672	0.33	0.302-1.495			
	>1500g	0.691	0.479	0.249-1.921			
**Resuscitation at birth**	Yes	1.65	0.326	0.607-4.487			
	No	1					
**Resuscitation in NICU**	Yes	3.033	0.013	1.260-7.302	2.773	0.086	0.867-8.874
	No	1					
**Chorioamnionitis**	Yes	0.5	0.541	0.054-4.611			
	No	1					
**PROM**	Yes	0.625	0.243	0.284-1.374	0.544	0.214	0.209-1.419
	No	1					
**Hypotension treatment**	None	1	–		1		
	Inotropes	–	–		–		
	NS bolus & inotropes	3.521	0.043	1.043-11.885	1.602	0.554	0.337-7.622
	NS bolus						
		5.03	0.07	0.879-28.793	2.662	0.353	0.337-21.002
**MV**	Yes	1.625	0.269	0.687-3.843			
	No	1					
**PDA**	Yes	1.763	0.175	0.776-4.002	0.56	0.27	0.200-1.567
	No	0.567	0.618	0.061-5.286			
	Unknown	1					
**Platelets**	<150000	1					
	>150000	0.577	0.143	0.276-1.205	0.65	0.392	0.242-1.743
**ANS**	Yes	0.062	0.012	0.007-0.537	0.075	0.028	0.007-0.757
	No	0.152	0.105	0.015-1.487	0.221	0.231	0.018-2.617
	Unknown	1					

**ANS, Antenatal steroids; MV, mechanical ventilation; PDA, Patent ductus arteriosus; PROM, Prolonged rupture of membranes**

### Short-term complications of IVH

Majority of the preterm neonates survived (82.2%) but there was still a notable mortality rate of 17.8%. Among the neonates with IVH, 26.6% (12) died, whereas only 13.3% (12) of preterm neonates without IVH succumbed to mortality. Survival rates were notably lower among neonates with IVH, with 73.3% (28) surviving compared to 86.6% (78) in preterm neonates without IVH. The incidence of PHVD was significantly higher among neonates with IVH, with 22.2% (10) developing hydrocephalus compared to 6.7% (6) in preterm neonates without IVH. Similarly, the occurrence of PVL was more prevalent in preterm neonates with IVH, with 20% (9) neonates affected, compared to 6.7% (6) in preterm neonates without IVH. Furthermore, one neonate (2.2%) among the preterm neonates with IVH developed simple cysts, while none were observed in preterm neonates without IVH Table 4.

**Table 4:** Frequency of short–term outcomes of IVH.

Complications	Any IVH (n = 45)	No IVH (n = 90)
**Dead**	12 (26.6%)	12 (13.3%)
**Alive**	33 (73.3%)	78 (86.6%)
**PHVD**	10 (22.2%)	6 (6.7%)
**PVL**	9 (20%)	6 (6.7%)
**Simple cysts**	1 (2.2%)	0

**PHVD, Post-hemorrhagic ventricular dilatation; PVL, Periventricular Leukomalacia**

## Discussion

IVH is the predominant cause of brain injury, posing significant challenges to neurodevelopment and impeding optimal brain growth, especially in babies born with a birth weight of less than 1500g or less than 32 weeks [10–12]. The increased number of preterm deliveries and their survival directly reflects the increased risk of IVH occurrence in these babies [4]. The few studies conducted in resource-limited settings show a wide-ranging incidence of IVH occurrence (8% − 34.2%) [14,15,24]. Implementation of small baby neuroprotective protocols, to help prevent IVH in some neonatal critical care units, has shown a reduction in the incidence of IVH [9]. This study was able to highlight the incidence of IVH in preterm babies, the associated risk factors and the short–term outcomes of IVH in our setting. We were able to see a great need to introduce a neuroprotective care bundle in order to reduce the incidence of IVH.

In this study, we observed a 33.3% incidence of IVH similar to the studies conducted in Uganda, Zambia and Kenya [14–16] and our incidence also falls within the global incidence range of 5–52% [5]. However, another Kenyan study conducted in KNH reported a lower IVH incidence of 8% [24]. The difference in the incidence may be attributed to the follow-up time noted to be upto 7 days of life vs 28 days of life in our study. Similar to this study, our unit also practices minimal patient handling and movement such as the use of a portal X-ray as opposed to moving the patient to the radiology department which has been reported to reduce the incidence of IVH occurrence.

The observed timing and severity of IVH in this study showed predominance in the mild grades of IVH particularly Grade I IVH with 10.4% occurring within the first 3 days of life followed by 15.6% occurrence by day 7 of life and 16.3% by day 28 of life. Our findings are similar to other studies that reported a predominance of the milder grades of IVH [14–16,24] with the majority occurring within the first 72 hours of life. This further depicts the importance of early screening and implementation of strategies to reduce this brain injury during this critical window.

The factors that showed a significant association with IVH occurrence in this study were resuscitation in NICU [28] and treatment of hypotension [29] with NS and inotropes. The need for resuscitation in the NICU and treatment of hypotension often alludes to an underlying severe illness, which may predispose preterm neonates to IVH. Hypotension causes fluctuations in cerebral blood flow whose autoregulatory function is impaired in preterm neonates. Rapid saline boluses increase the cerebral blood flow causing injury to the fragile germinal matrix blood vessels and thus further increasing the risk of IVH [30]. Our findings are similar to Khanafer-Larocqu et al, who reported an increased incidence of IVH with the use of saline boluses and inotropes. Khanafer-Larocqu et al, also observed that treatment of a hemodynamically significant PDA was associated with a higher risk of IVH. This increased risk was also noted in our study however it was not statistically significant, an observation that could be due to the smaller sample size. Maternal exposure to antenatal steroids was confirmed to have a protective role in the prevention of IVH development in our study. Antenatal steroid administration is known to enhance lung maturity and reduce the risk of IVH by stabilizing cerebral blood vessels in preterm neonates. This finding reinforces the importance of antenatal corticosteroid therapy in high-risk pregnancies and supports ongoing efforts to ensure the timely administration of corticosteroids [15,16,30,31]. Majority of the deliveries occurred in AKUHN stressing the need to deliver this high-risk group of neonates in a specialized center that can take care of them and prevent adverse outcomes like brain injury. Our findings on the mode of delivery via cesarian section are similar to other studies that found it to be protective against IVH development [15,24,31–33]. This finding might be attributable to the greater stress experienced during vaginal delivery, which could impact cerebral hemodynamics. Treatment with MV, having a hemodynamically significant PDA and a low platelet count of less than 150000 showed an increased risk of IVH occurrence however this risk did not reach a significant association with IVH occurrence as reported in other studies [24,30]. Many studies have shown an association of these factors with an increased risk of IVH as the birth weight and gestation age decrease [14,15,17,24,32–34]. We noted an increase in the incidence of IVH with a decline in birth weight and gestation although this failed to show a significant association. Maternal risk factors like Chorioamnionitis and PROM presented as protective risk factors in our study however this association didn’t reach statistical significance. Chorioamnionitis as a prenatal exposure following PROM causes increased production of inflammatory markers like cytokines causing damage to the blood-brain barrier resulting in influx of plasma-protein and oligodendroglia damage [35].

The incidence of death was notably higher in neonates with IVH (26.6%) compared to those without IVH (13.3%). This difference is indicative of a significant association between IVH and increased mortality. This finding aligns with existing literature indicating that severe IVH is often associated with higher mortality rates due to its impact on neurological and overall health [15,33]. The increased risk of death in neonates with IVH highlights the need for intensive monitoring and management to improve survival outcomes. PHVD was present in 22.2% of preterm neonates with IVH compared to only 6.7% in those without IVH. IVH can lead to PHVD due to obstruction of cerebrospinal fluid (CSF) pathways or impaired CSF absorption [36]. The higher prevalence of PHVD in the IVH group highlights the need for close follow-up and potential interventions to manage and mitigate the risk of developing this complication. PVL was observed in 20% of preterm neonates with IVH compared to 6.7% in those without IVH. Our findings could be higher than the study done in Uganda [15] which reported 2.4% of cystic PVL cases because MRI studies were performed as a follow-up investigation for preterm neonates with IVH on day 28 of life.

Notably, the design of the study being retrospective brings with it limitations like missing data. For instance, a lack of comprehensive data on all the factors of interest as it relates to IVH occurrence, such as, delayed cord clamping, that has been shown to have a protective role in reducing the risk of IVH. Additionally, we didn’t have sufficient information to generate the SNAPPE scores to predict the risk of mortality in our study participants. The small sample size could also have influenced the failure to see an association between some of the established risk factors. Nonetheless, this study has been able to assess the role of the most common factors linked to IVH in the existing literature within our setting. It is also important to highlight that the study participants were drawn from a single health facility which raises concerns regarding the generalizability of study findings, however, as previously described AKUHN is a major regional referral center receiving high risk babies not just from within the institution but across East and Central Africa strengthening the external validity of our study findings.

In this regard, this study represents one of the few studies undertaken in East and Central Africa representing the magnitude of IVH in this high-risk group. The availability of cranial US scans that were performed, reported and validated by a Paediatric radiologist, are a major strength of this study. Also, in contrast to previous research [24], we were also able to extend the IVH screening time to 28 days of life and report the short-term outcomes of IVH. This is important, as most of the preterm babies discharged before 28 days of life in our setting are either lost to follow-up or miss having their 28-day ultrasound scan done.

## Conclusion

The findings elicited from this study as discussed above have provided baseline data on the incidence of IVH and some of the associated risk factors in this highly vulnerable age group. The latter lays a foundation for future studies aimed at validating existing neuroprotective protocols currently only available in developed countries which carry the potential to reduce the magnitude of IVH. In turn, these neuroprotective protocols help these babies not only to survive but also thrive as we reduce the adverse outcomes that may occur with brain injury.
